# De Novo Psoriasis Vulgaris Diagnosed after Nivolumab Treatment for Refractory Hodgkin's Lymphoma, Completely Resolved after Autologous Hematopoietic Stem Cell Transplantation

**DOI:** 10.1155/2018/6215958

**Published:** 2018-10-16

**Authors:** Panayotis Kaloyannidis, Eshrak Al Shaibani, Miral Mashhour, Mohammed Gamil, Ioannis Apostolidis, Hani Al Hashmi, Khalid Ahmed Al Anazi

**Affiliations:** ^1^Adults Hematology and Stem Cell Transplantation Department, King Fahad Specialist Hospital, Dammam, Saudi Arabia; ^2^Department of Pathology and Laboratory Medicine, King Fahad Specialist Hospital, Dammam, Saudi Arabia; ^3^Dermatology Department, King Fahad Specialist Hospital, Dammam, Saudi Arabia

## Abstract

The programmed cell death protein-1 (PD-1) inhibitor nivolumab has been recently approved as an effective and safe treatment for patients with refractory/relapsed Hodgkin's lymphomas. Dermatological adverse events, mainly skin rash, have been reported in 1–5% of patients. We describe a case of de novo psoriasis vulgaris (PsV), diagnosed after nivolumab treatment for refractory Hodgkin's lymphoma. After administration of 6 cycles, skin lesions appeared in the right tibia, forearms, and dorsum of hands, and biopsy confirmed the diagnosis of PsV. The lesions completely resolved after autologous stem cell transplantation (ASCT) which was performed in the context of the treatment for the primary disease. PsV is an inflammatory skin disease, and it is considered to be mediated through cytotoxic T-cells. PD-1 blockage may lead to expansion of such T-cells, resulting thus in PsV appearance. The early published studies showed that nivolumab represents a safe treatment approach. PsV occurrence has not been reported so far in patients treated with nivolumab for hematological diseases, and it seems that long-term follow-up is necessary to fully clarify the entirety of PD-1 inhibitors' skin adverse events. Additionally, this clinical observation provides an evidence for a potential exploitation of ASCT in refractory and severe forms of PsV.

## 1. Introduction

The blockade of immunosuppressive pathways, also known as “immune checkpoints,” represents an innovative treatment approach for patients with refractory solid and hematological malignancies [1–4]. Nivolumab (Opdivo, Bristol-Myers Squibb Company), a monoclonal antibody against programmed cell death protein-1 (PD-1), has shown extremely promising results with durable disease responses, and in May 2016, it was approved by the U.S. Food and Drug Administration for the treatment of refractory Hodgkin's lymphoma patients [5]. Given the mechanism of action that triggers T-cell activation, nivolumab may induce specific adverse events (AEs), including immune-related cutaneous toxicities. In the published clinical trials, the cutaneous complications among patients with hematological diseases are reported to be nonspecific, such as macular papular rash and pruritus, which usually are readily manageable [2–6].

We herein describe a rare dermatologic AE psoriasis vulgaris (PsV) that appeared in a patient who was treated with nivolumab for refractory classical Hodgkin's lymphoma (cHL).

## 2. Case Report

**Figure 1:**
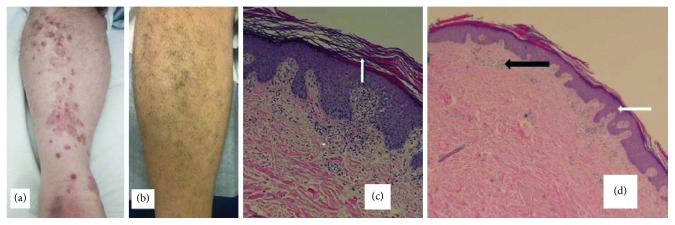
Skin lesions and skin biopsy with hematoxylin-eosin stain. (a) After 6 cycles of nivolumab and before ASCT: scaly erythematous annular plaque on the right tibia. (b) Twelve months after ASCT: skin lesions completely disappeared. (c) Skin biopsy: epidermis lesions showing hyperkeratosis. (d) Skin biopsy: lesions showing acanthosis (white arrow) and perivascular infiltration of mononuclear cells indicating chronic inflammation (black arrow).

A 53-year-old man who was firstly treated for diffuse large B-cell lymphoma (DLBCL) presented 45 months after induction remission treatment with abdominal and inguinal lymph node (L/N) enlargement. An excisional L/N biopsy confirmed the histological type of mixed cellularity cHL; malignant cells were positive for CD30, CD15, and PAX5 and negative for CD20, CD10, CD3, BCL-2, and EMA antigens. ESHAP (etoposide, cisplatin, methylprednisolone, and cytarabine) was given as salvage treatment, and after 2 cycles, he achieved very good partial remission. Since the treatment plan was to proceed with high-dose chemotherapy and rescue with autologous stem cells transplantation (ASCT), he received an additional 3^rd^ cycle of ESHAP for further disease control and autologous stem cell collection. After the 3^rd^ cycle of salvage chemotherapy, the disease further responded and the stem cells collection was successful. However, he developed acute kidney injury, and the ASCT postponed till renal function recovery; the patient, based on his previous medical history (DLBCL and cHL diagnoses), received a combination of rituximab plus brentuximab vedotin as bridge treatment to ASCT. Four months later, the renal function became normal, but evaluation with PET-CT (after six cycles of combination treatment) confirmed disease progression. Subsequently, nivolumab at the dose of 3 mg/m^2^ every two weeks was given as a new salvage therapy. The medication was well tolerated, and no renal or any other organ function impairment was noticed. However, after the sixth infusion of nivolumab, he presented with raised nonitchy, erythematous scaly papules with silver-white coating and some annular plaques with collarettes of scales of different sizes involving the anterolateral aspects of shins and dorsa of hands, distal forearms, and both tibias (Figure 1). The Köbner phenomenon was not noticed. The clinical differential diagnosis included (1) PsV, (2) erythema annulare, or (3) tinea circinata. To confirm the diagnosis, a 5 mm punch skin biopsy was obtained from the right tibia. The epidermis findings revealed hyperkeratosis and irregular acanthosis with regular elongation of rete ridges and suprapapillary thinning (Figures 1 and 1). The upper dermis showed mild perivascular lymphocytic infiltration (Figure 1). There were no evidence of Munro microabscesses and no evidence of granuloma or other specific inflammation. Although the histological findings were not the typical one, the diagnosis of PsV was made based on the clinicopathological findings and the exclusion of the other aforementioned dermatological diseases.

The patient initially received only topical treatment with steroids, and the skin lesions partially improved. He next underwent ASCT, with a conditioning regimen consisted of single-agent chemotherapy of melphalan 200 mg/m^2^. The skin lesions gradually improved, and 3 months after ASCT, they almost disappeared. Currently, one year after ASCT, the patient was alive in complete metabolic remission regarding his primary disease and without any evidence of residual skin lesions (Figure 1).

## 3. Discussion

Nivolumab, although it belongs to the “monoclonal antibodies family” agents, unlikely to other monoclonal antibodies, directly triggers the T-cell activation and therefore is associated with a specific toxicity profile and adverse events that are mostly of immune origin.

In a series of patients treated with nivolumab for solid malignant diseases or lymphomas, cutaneous reactions were not uncommon and mainly concerned skin rash, pruritus, and vitiligo [2–7]. Although the exacerbation of previously diagnosed PsV is a well-described phenomenon after PD-1 inhibitor treatment, the *de novo* appearance of PsV is uncommon, and only few sporadic cases have been described so far, all of them in patients with nonhematological cancers [8–12]. Among patients with cHL who were treated with nivolumab, although skin reactions have been noticed, drug-induced psoriasis never has been reported, at least to our best knowledge [2–8, 13].

PsV is a chronic inflammatory skin disease with a complex autoimmune etiology, and it is considered to be mediated by cytotoxic T-cells and more specifically by T-cells polarized to a Th1 and Th17 fate [14]. PD-1 inhibitors have been demonstrated to augment Th1 and Th17 cell activities, and by these immunological consequences, nivolumab has been potentially implicated in either PsV new occurrence or PsV reactivation [15]. We assume that in our heavily pretreated patient, the exposure to nivolumab (even for short tem period), was quite enough to trigger residual T cells which subsequently targeted antigens on the dermis/epidermis cells resulting thus in PsV appearance.

The treatment of PsV still remains challenging, and several topical and systemic treatments (including monoclonal antibodies and immunomodulating factors) have been used and proposed so far [16, 17].

Regarding the correlation of PD-1 inhibitors treatment and PsV, Bonigen et al. published recently a review study, the largest so far, of 21 patients with reactivated or newly diagnosed PsV after PD-1 inhibitors treatment . All patients had diagnosis exclusively of solid tumor (melanoma or lung cancer). Fourteen out of 21 had been previously treated with nivolumab, and 12 of them showed improvement after local (7 patients) or systemic (5 patients) treatment. However, it is not reported whether complete resolution of PsV occurred in any patient [8].

In our case, the skin lesions mildly improved after local steroids treatment and finally resolved almost completely within 3 months after ASCT. It is well known that the symptoms of several autoimmune diseases, including psoriasis disease, can be controlled and minimized after high-dose chemotherapy followed by allogeneic or autologous stem cells rescue [18]. However, the experience of ASCT in psoriasis with only skin involvement, especially in PsV form, is extremely limited, and only sporadic cases have been reported [19–22]. We speculate that, in our case, the conditioning regimen consisted of 200 mg/m^2^ melphalan followed by autologous stem cell infusion facilitated the process of the complete PsV resolution.

PD-1 inhibitors have been only recently introduced in physicians' “pharmaceutical quiver” for the treatment of advanced hematological diseases. Although the early published studies showed only mild and easily manageable immune skin reactions, it seems that long-term follow-up is necessary to clarify the entirety of PD-1 inhibitors' skin adverse events. On the contrary, given its mechanism of action and its current low toxicity profile, ASCT could be studied in clinical trials as a potential therapeutic approach for patients with a severe and refractory form of PsV.
